# Supplementary material for: HIVprotI: an integrated web based platform for prediction and design of HIV proteins inhibitors
Source: J Cheminform. 2018 Mar 9;10:12. doi: 10.1186/s13321-018-0266-y (PMC5845081; doi:10.1186/s13321-018-0266-y)
Supplement: Supplementary file 2 — Additional file 2. Source code of HIVProtI web server. [file 13321_2018_266_MOESM2_ESM.zip › HIVprotI_Source-code/hivprotI/weka-3-6-6/doc/weka/classifiers/trees/j48/NBTreeClassifierTree.html]

NBTreeClassifierTree


---


|  |  |  |
| --- | --- | --- |
| **Overview** \| **Package** \| **Class** \| **Tree** \| **Deprecated** \| **Index** \| **Help** \| **Weka's home** | |  |
| **PREV CLASS**   **NEXT CLASS** | **FRAMES**    **NO FRAMES**     **All Classes** |  |
| SUMMARY: NESTED \| FIELD \| CONSTR \| METHOD | DETAIL: FIELD \| CONSTR \| METHOD |  |


---


## weka.classifiers.trees.j48 Class NBTreeClassifierTree

```
java.lang.Object
  weka.classifiers.trees.j48.ClassifierTree
      weka.classifiers.trees.j48.NBTreeClassifierTree
```

**All Implemented Interfaces:**: java.io.Serializable, CapabilitiesHandler, Drawable, RevisionHandler

---

``` public class NBTreeClassifierTree extends ClassifierTree ```

Class for handling a naive bayes tree structure used for
classification.

**Version:**
:   $Revision: 5535 $

**Author:**
:   Mark Hall (mhall@cs.waikato.ac.nz)

**See Also:**: Serialized Form

---

**Field Summary**

**Fields inherited from interface weka.core.Drawable**

`BayesNet, Newick, NOT_DRAWABLE, TREE`


**Constructor Summary**

`NBTreeClassifierTree(ModelSelection toSelectLocModel)`


| **Method Summary** | |
| --- | --- |
| `void` | `buildClassifier(Instances data)`             Method for building a naive bayes classifier tree |
| `Capabilities` | `getCapabilities()`             Returns default capabilities of the classifier tree. |
| `java.lang.String` | `getRevision()`             Returns the revision string. |
| `java.lang.String` | `graph()`             Returns graph describing the tree. |
| `java.lang.String` | `printLeafModels()`             Print the models at the leaves |
| `java.lang.String` | `toString()`             Prints tree structure. |

**Methods inherited from class weka.classifiers.trees.j48.ClassifierTree**

`assignIDs, buildTree, buildTree, classifyInstance, cleanup, distributionForInstance, graphType, numLeaves, numNodes, prefix, toSource`

**Methods inherited from class java.lang.Object**

`equals, getClass, hashCode, notify, notifyAll, wait, wait, wait`

**Constructor Detail**

### NBTreeClassifierTree

```
public NBTreeClassifierTree(ModelSelection toSelectLocModel)
```


**Method Detail**

### getCapabilities

```
public Capabilities getCapabilities()
```

:   Returns default capabilities of the classifier tree.

    :   **Specified by:**: `getCapabilities` in interface `CapabilitiesHandler` **Overrides:**: `getCapabilities` in class `ClassifierTree`
    :   **Returns:**: the capabilities of this classifier tree **See Also:**: `Capabilities`

---


### buildClassifier

```
public void buildClassifier(Instances data)
                     throws java.lang.Exception
```

:   Method for building a naive bayes classifier tree

    :   **Overrides:**: `buildClassifier` in class `ClassifierTree`
    :   **Parameters:**: `data` - the data to build the tree from **Throws:**: `java.lang.Exception` - if something goes wrong

---


### printLeafModels

```
public java.lang.String printLeafModels()
```

:   Print the models at the leaves

    :   **Returns:**: textual description of the leaf models

---


### toString

```
public java.lang.String toString()
```

:   Prints tree structure.

    :   **Overrides:**: `toString` in class `ClassifierTree`
    :   **Returns:**: the tree structure

---


### graph

```
public java.lang.String graph()
                       throws java.lang.Exception
```

:   Returns graph describing the tree.

    :   **Specified by:**: `graph` in interface `Drawable` **Overrides:**: `graph` in class `ClassifierTree`
    :   **Returns:**: the tree as graph **Throws:**: `java.lang.Exception` - if something goes wrong

---


### getRevision

```
public java.lang.String getRevision()
```

:   Returns the revision string.

    :   **Specified by:**: `getRevision` in interface `RevisionHandler` **Overrides:**: `getRevision` in class `ClassifierTree`
    :   **Returns:**: the revision


---


|  |  |  |
| --- | --- | --- |
| **Overview** \| **Package** \| **Class** \| **Tree** \| **Deprecated** \| **Index** \| **Help** \| **Weka's home** | |  |
| **PREV CLASS**   **NEXT CLASS** | **FRAMES**    **NO FRAMES**     **All Classes** |  |
| SUMMARY: NESTED \| FIELD \| CONSTR \| METHOD | DETAIL: FIELD \| CONSTR \| METHOD |  |


---
